# Identification and Characterization of Extrachromosomal Circular DNA in Slimming Grass Carp

**DOI:** 10.3390/biom14091045

**Published:** 2024-08-23

**Authors:** Haobin He, Zihan Gao, Zehua Hu, Guanyu Liang, Yanhua Huang, Meng Zhou, Rishen Liang, Kai Zhang

**Affiliations:** 1 Innovative Institute of Animal Healthy Breeding, Zhongkai University of Agriculture and Engineering, Guangzhou 510225, China; hehaobin@zhku.edu.cn (H.H.); gaozihan@zhku.edu.cn (Z.G.); huzehua@zhku.edu.cn (Z.H.); liangguanyu@zhku.edu.cn (G.L.); huangyanhua@zhku.edu.cn (Y.H.); zhoumeng@zhku.edu.cn (M.Z.); 2 College of Animal Science and Technology, Zhongkai University of Agriculture and Engineering, Guangzhou 510225, China; 3 College of Life Sciences and Oceanography, Shenzhen University, Shenzhen 518060, China

**Keywords:** extrachromosomal circular DNA, slimming grass carp, muscle characteristic

## Abstract

Slimming grass carp is a commercial variety with good body form and meat quality, which is cultured by starving common grass carp in a clean flowing water environment. Compared to common grass carp, slimming grass carp has a far higher economic value. Until now, no molecular study has concentrated on the regulation mechanism of the muscle characteristics of slimming grass carp. This study first reported the gene expression profile of the muscle characteristics of slimming grass carp based on the level of extrachromosomal circular DNAs (eccDNAs). EccDNAs are double-stranded circular DNAs derived from genomic DNAs and play crucial roles in the functional regulation of a wide range of biological processes, none of which have been shown to occur in fish. Here, muscle eccDNAs from slimming grass carp and common grass carp were both generally sequenced, and the information, as well as the expression profile of eccDNAs, were compared and analysed. The findings reveal that 82,238 and 25,857 eccDNAs were detected from slimming grass carp and common grass carp, respectively. The length distribution of eccDNAs was in the range of 1~1000 bp, with two peaks at about 200 bp and 400 bp. When the expression profiles of eccDNAs between slimming grass carp and common grass carp were compared, 3523 up-regulated and 175 down-regulated eccDNAs were found. Enrichment analysis showed that these eccDNA genes were correlated with cellular structure and response, cell immunology, enzyme activity, etc. Certain differentially expressed eccDNAs involved in muscle characteristics were detected, which include myosin heavy chain, myosin light chain, muscle segment homeobox C, calsequestrin, calmodulin, etc., among which the majority of genes were linked to muscle structure and contraction. This indicates that during the process of cultivating from common grass carp to slimming grass carp, the treatment primarily affected muscle structure and contraction, making the meat quality of slimming grass carp different from that of common grass carp. This result provides molecular evidence and new insights by which to elucidate the regulating mechanism of muscle phenotypic characterisation in slimming grass carp and other fish.

## 1. Introduction

Grass carp (*Ctenopharyngodon idellus*) is one of the most important aquaculture species and is highly cultivated and consumed in China. More than 5.7 million tonnes of grass carp are produced in China alone, accounting for one-fifth of all freshwater aquaculture production [1]. Due to inadequate feed nutrition, irrational feeding, and water pollution, the meat quality of grass carp has seriously declined in recent years, which greatly limits its use in the field of aquaculture and processing [2]. In an effort to improve the meat quality and commercial value of grass carp, researchers have developed a slimming cultivation method for this species. The cultivation method of slimming grass carp involves placing common adult grass carp with fat bellies into clean flowing water without feeding for a period of time. This reduces the weight and fat and transforms the grass carp into fish with a good body shape, firm meat, delicious taste, and a faint fragrance that does not smell like mud [3]. The economic value of slimming grass carp is much higher than that of common grass carp, which significantly aids in the development of grass carp cultivation. Recently, there have been very few studies on slimming grass carp; some reports have only concentrated on cultivation technology and marketing [4], with some focusing on the nutritional quality of muscle [5,6,7]. Zhou Bin et al. [7] revealed that fat decreased in grass carp after slimming cultivation, but protein and mineral content increased, along with an increase in muscular hardness. Nevertheless, no study has examined the molecular mechanism behind the change in the muscle characteristics of slimming grass carp relative to common grass carp. After slimming treatment, what kind of changes existed in grass carp muscle? Which genes present differential expression? Which genes are involved in the functional regulation? There is no information available to answer these questions.

Extrachromosomal circular DNAs (eccDNAs) are double-stranded circular DNA derived from chromosomal DNA and are independent of the extrachromosomal genome [8]. They were stable and less susceptible to nuclease degradation than chromosomal DNA [9]. EccDNAs are commonly found in eukaryotes, and they range in size from a few hundred to a million bases [10,11,12,13,14]. They usually carry partial or complete genes, as well as functional elements, and participate in the occurrence and development of senescence, gene compensation, drug resistance, and tumourigenesis [15,16,17,18,19]. The circular DNA molecule outside the chromosome in pig sperm was first identified by Hotta and Bassel [20]. Later, Cox discovered many free chromosomes of varying sizes outside of the chromosome in neurotumour cells [9]. Radloff et al. compared the circular DNA in the Hela nucleus with mitochondrial DNA and pointed out that the length of the nucleus circular DNA was 0.2–19.8 µm, while the length was (4.81 ± 0.24) µm in mitochondrial DNA. Such detection further verified the existence of circular DNA in eukaryotic cells, with the exception of mitochondrial DNA [21]. Currently, most studies on eccDNA are focused on human tumours and cancers, and their findings have shown that eccDNA can regulate the progression of tumours by increasing gene copy numbers [15,16,17]. In recent years, some studies also started to analyse the functional regulation of animal breeding based on eccDNA level and have demonstrated a significant role of eccDNA in regulating animal phenotypic traits [22,23]. However, no eccDNA studies have been reported on fish, and all information was unknown.

In this study, we first determined the eccDNAs of slimming grass carp and common grass carp based on the next-generation sequencing method. Then, we compared and analysed the content differences of eccDNAs in the two kinds of fish muscle cells and examined the differential expressions and regulatory roles of eccDNA on the change in muscle quality. This study aimed to provide a new methodology and potential reference for elucidating the regulation mechanism of fish muscle characteristics based on the eccDNA level. 

## 2. Material and Method

### 2.1. Samples Collection and DNA Extraction

Two groups of grass carp, slimming grass carp (*n* = 5) and common grass carp (*n* = 5), obtained from the aquatic farm of Qingyuan City, Guangdong Province, were selected for this study. The slimming grass carp had been placed into the clean flowing water without feeding for two months, while common grass carp were cultivated in normal pattern with ordinary feeding. Partial back muscles of each sample were cut and transferred into liquid nitrogen, which was then kept at −80 °C. Total genomic DNA was extracted from about 100 mg muscle tissue using a MagAttract HMW DNA Kit (Qiagen, Frankfurt, Germany), and it was examined using agarose gel electrophoresis. The extracted DNA content was measured using a NanoDrop Microvolume Spectrophotometer (Thermo Scientific, Waltham, MA, USA). 

### 2.2. Linear DNA Removal and Circular DNA Enrichment

After examination, the extracted DNA of 5 slimming grass carp and 5 common grass carp samples were continuously digested at 37 °C for 120 h by Plasma-SafeTM ATP-dependent DNase (Epicentre, E3110K, Madison, WI, USA) to remove linear chromosomal DNA. According to the method of current eccDNA studies [24,25], two genes—cytochrome-c oxidasesubunit III (*COX3*) and nudix (nucleoside diphosphate-linked moiety X)-type motif 6 (*Nudt6*)—were used for detecting the effect of linear chromosomal DNA removal. *COX3* is a gene from mitochondrial DNA, which like eccDNA, is also a double-stranded circular DNA, while *Nudt6* is a gene from linear chromosomal DNA. When the specific bands of our samples were detected in the *COX3* gene and no band in the Nudt6 gene via electrophoresis after PCR amplification, the linear chromosomal DNA was completely removed. The primers information of the two genes were as follows: *COX3*-F: 5′-GGC CAT CCA ATC TCT CGC AC-3′, *COX3*-R:5′-TGA CGT GTA GTC CGT GGA ATC C-3′; *Nudt6*-F: 5′-GTA GAG CTA TTG CGT GTC TCG A-3′; *Nudt6*-R: 5′-GAC AGT CTT TCT TGC AGC ATG G-3′. Additionally, one randomly selected genomic DNA sample incubated at 37 °C for 120 h without digestion of Plasma-SafETM ATP-dependent DNase was used as a positive control (PC) to evaluate the influence of DNA quality under the treatment.

The PCR amplification condition of *COX3* and *Nudt6* were as follow: Initial denaturation at 94 °C for 5 min, followed by 35 cycles with 94 °C for 30 s, 55 °C (*COX3*) or 62 °C (*Nudt6*) for 30 s and 72 °C for 60 s, final extension at 72 °C for 5 min. PCR products were identified by agarose gel electrophoresis. After removing linear genomic DNA, circular DNA were randomly amplified using a Phi29 polymerase and exo-resistant random primers via rolling circle amplifcation (RCA). RCA reaction was performed at 30 °C for 72 h. The enriched eccDNA was isolated using Cycle-pure Kit purifcation (Omega, Norcross, GA, USA) and then digested with NdeI (Thermo Scientific, Waltham, MA, USA) to verify the RCA result prior to sequencing. 3 samples of each slimming grass carp and common grass carp with good extraction quality were selected for the eccDNA next-generation sequencing.

### 2.3. Illumina Sequencing

The obtained eccDNA-enriched DNAs were sheared via sonication (Covaris, Inc. Woburn, MA, USA), and fragmented DNA was extracted for DNA library construction using Illumina (New England Biolabs, Ipswich, MA, USA) NEBNext DNA Library Prep Kit. Sequencing of 150 bp paired-end patterns was performed on an Illumina NovaSeq 6000 in accordance with the manufacturer’s instructions.

### 2.4. Data Analysis

Raw read data were obtained from the Illumina NovaSeq 6000 sequencer. Trimmomatic software (v0.32) [26] was used to filter all of the data in order to remove low-quality reads. The overall quality of the raw reads was then evaluated using Q30 as the QC standard to confirm the good sequencing quality. Burrows–Wheeler Aligner (BWA) software (v0.7.17) [27] was used to align the high-quality clean reads to the whole grass carp genome sequence (GCF_019924925) from NCBI. After that, the eccDNAs were detected using the circular DNA analysis software Circle-Map (v1.1.4) [28] and filtered with the parameters (split ≥ 1) to improve the accuracy. Annotation of the eccDNAs was conducted using bedtools software (v2.31.0) [29]. Functional profiling of eccDNAs was carried out using gene ontology (GO) and Kyoto encyclopedia of genes and genomes (KEGG) pathway enrichment analyses based on differentially expressed eccDNA-related genes. IGV (v2.4.10) software was employed to visualise eccDNAs.

### 2.5. Statistical Analysis

Statistical descriptions and analyses were carried out using SPSS 26.0 statistical software with a = 0.05 as the test level. All results are expressed as the mean ± standard deviation. The difference between the two groups was checked using the independent sample *t*-test. *p* < 0.05 was deemed statistically significant.

## 3. Results

### 3.1. Detection of Linear DNA Removal and Circular DNA Enrichment

**Figure 1:**
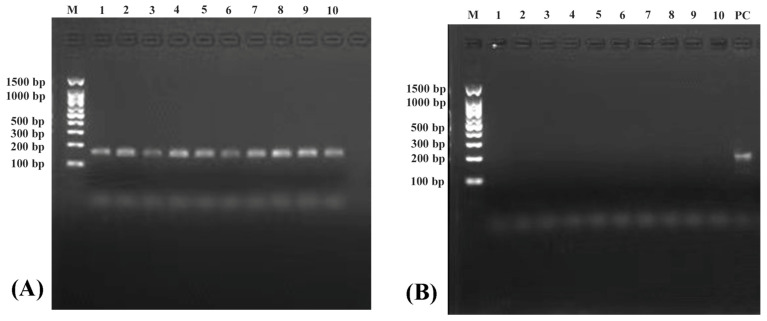
Electrophoresis results in the amplification of *COX3* (**A**) and *Nudt6* (**B**) genes. Numbers 1~5 represent slimming grass carp samples; numbers 6~10 represent common grass carp samples; PC represents the positive control sample.

The electrophoresis results of *COX3* and *Nudt6* genes amplification from five slimming grass carp and five common grass carp are shown in Figure 1A,B, respectively. A specific band was detected in the *COX3* gene, while no band was detected in *Nudt6*. Moreover, a significant band of the *Nudt6* gene was still detected in PC sample, indicating that linear chromosomal DNA was completely removed and circle DNA was retained.

**Figure 2:**
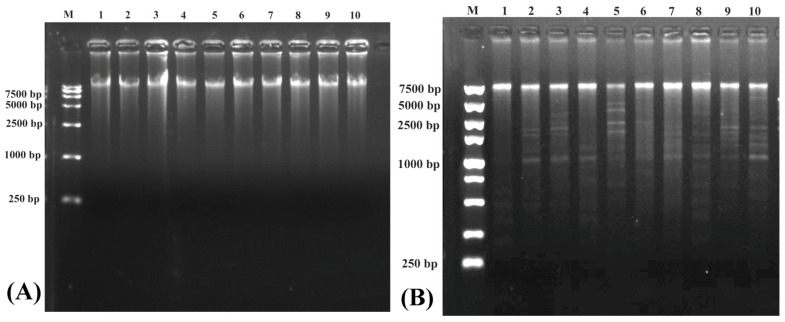
Electrophoresis results in the purification of eccDNAs after RCA reaction (**A**) and enzyme digestion verification (**B**). Numbers 1~5 represent samples of the slimming grass carp; 6~10 represent samples of the common grass carp.

Electrophoresis detection in the purification of eccDNAs after RCA reaction is shown on Figure 2A. Significant bands were found in each sample, revealing that the whole eccDNAs were successfully enriched by the RCA reaction. The EccDNAs of all samples can be digested to be fragments in different sizes (Figure 2B), indicating that enriched eccDNAs were intact and in good quality.

### 3.2. The Lansacape of eccDNA

The original reads (raw reads) were obtained from the Illumina NovaSeq sequencer and filtered using Trimmomatic software. After filtering, the overall quality of the raw reads was evaluated using Q30 as the QC standard. A Q30 > 80% indicates good sequencing quality. BWA software (v0.7.17) was used to align the high-quality clean sequences to the whole grass carp genome. The number of clean reads for slimming grass carp (1, 2, 3) and common grass carp (1, 2, 3) were 130,405,698, 164,979,168, and 125,514,344 and 139,871,412, 123,377,174, and 130,405,698, respectively (Table 1).

**Figure 3:**
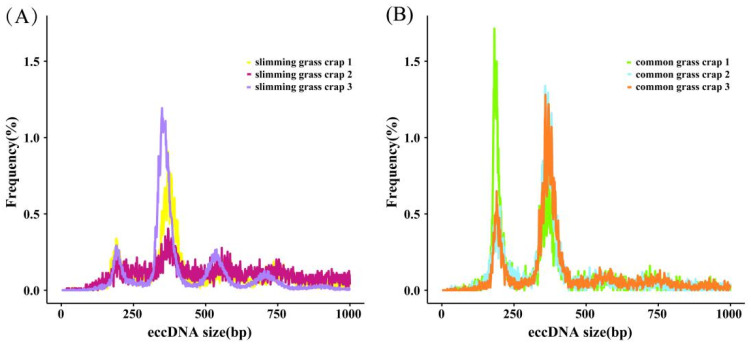
The lengths distribution of eccDNAs in slimming grass carp (**A**) and common grass carp (**B**).

Amounts of 82,238 and 25,857 eccDNAs were detected in the slimming grass carp and common grass carp, respectively. Through the annotation and classification of these eccDNA, we found that all gene types were mRNA. The length distribution of eccDNA in the grass carp samples was wide, ranging from 1 bp to 1000 bp, but they mainly concentrated in two peaks at about 200 bp and 400 bp. In samples of common grass carp, the length distributions of the eccDNAs were both concentrated at 200 bp and 400 bp, while in slimming grass carp, they were mainly concentrated at 400 bp; another small peak at about 550 bp was also found in slimming grass carp, indicating that eccDNAs are prevalent in both common grass carp and slimming grass carp, but slimming grass carp are prone to produce relatively longer eccDNAs than common grass carp, which enable them to carry longer functional genomic segments (Figure 3).

**Figure 4:**
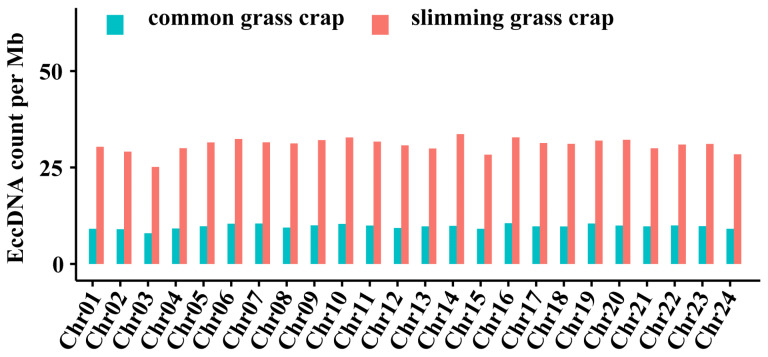
The distribution of eccDNAs in different chromosomes.

Muscle eccDNAs were widely distributed across all 24 chromosomes. However, upon analysing the frequency of eccDNAs per Mb on each chromosome, we found that in slimming grass carp, the frequency of eccDNAs in most chromosomes was 25~35 per Mb, whereas the frequency was mostly below 10 per Mb in common grass carp. This reveals that the numbers of eccDNA derived from each chromosome in slimming grass carp were higher in comparison to those in common grass carp. Nonetheless, the number of eccDNAs in each chromosome showed little variation. In slimming grass carp, the highest eccDNA number was derived from chromosome 14, followed by chromosome 16, and the lowest eccDNA number was from chromosome 3 (Figure 4).

### 3.3. GC Content Distribution near EccDNA Sequence

**Figure 5:**
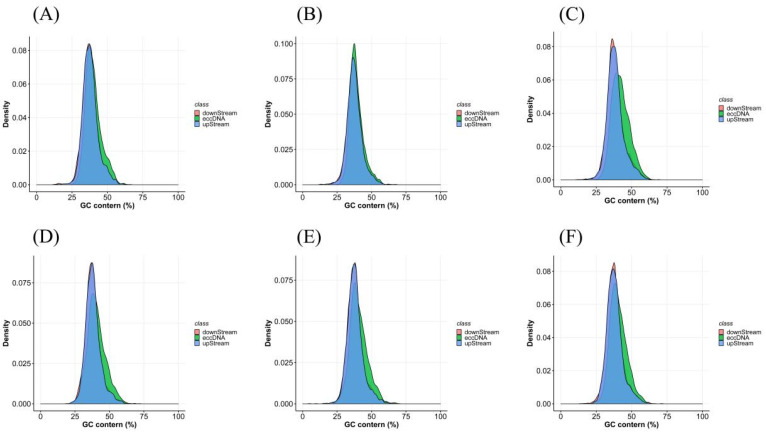
The GC content of eccDNA in slimming grass carp (**A**–**C**) and common grass carp (**D**–**F**).

By examining the GC content in the eccDNA region and the 1000 bp upstream and 1000 bp downstream regions of the connection point, we found that there was considerable overlap in the GC content in all three regions. All grass carp samples had content in the range of 25~60%, with a peak value at about 35% (Figure 5A–F).

### 3.4. GO Analysis

**Figure 6:**
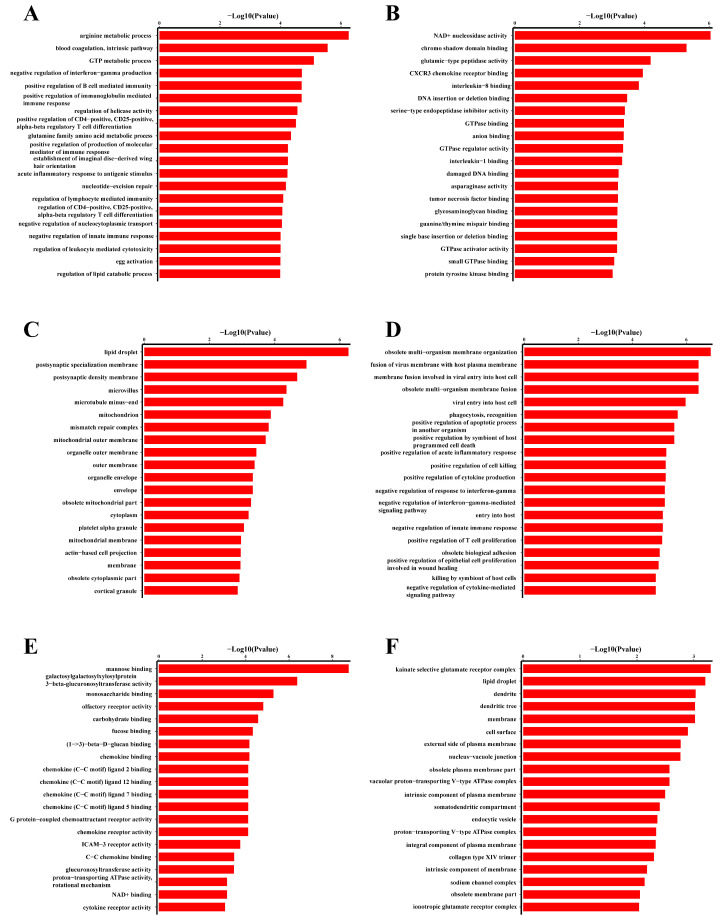
GO analysis of differentially expressed eccDNA-associated mRNAs: (**A**–**C**) refer to the GO enrichment of up-regulated eccDNAs in slimming grass carp in comparison to common grass carp; (**D**–**F**) refer to the GO enrichment of down-regulated genes in slimming grass carp in comparison to common grass carp.

To examine the potential biological functions of eccDNAs in the muscle characteristic of slimming grass carp further by comparing them with common grass carp, we conducted a GO enrichment analysis of eccDNAs between groups using the “Clusterprofiler (v4.12.3)” R package with the criteria of *p*-value < 0.05 and |log2 FC| ≥ 1. Differently expressed genes were classified into three major categories: biological process (BP); cellular component (CC); and molecular function (MF) The most enriched GO terms of downregulated and upregulated eccDNAs are shown in Figure 6. For the genes associated with the up-regulated eccDNAs, the first three significant enriched terms of BP were “arginine metabolic process (GO:0006525)”, “blood coagulation, intrinsic pathway (GO:0007597)”, and “GTP metabolic process (GO:0046039)”. The first three enriched terms of CC were “NAD+ nucleosidase activity (GO:0003953)”, “chromo shadow domain binding (GO:0070087)”, and “glutamic−type peptidase activity (GO:0070002)”. The first three enriched terms of MF were “lipid droplet (GO:0005811)”, “postsynaptic specialization membrane (GO:0099634)”, and “postsynaptic density membrane (GO:0098839)” (Figure 6A–C). For the genes corresponding to the down-regulated eccDNAs, the first three enriched terms of BP were “obsolete multi-organism membrane organization (GO:0044803)”, “fusion of virus membrane with host plasma membrane (GO:0019064)”, and “membrane fusion involved in viral entry into host cell (GO:0039663)”. The first three enriched terms of CC were “mannose binding (GO:0005537)”, “Galactosylgalactosylxylosylprotein 3-beta-glucuronosyltransferase activity (GO:0015018)”, and “monosaccharide binding (GO:0048029)”. The first three enriched terms of MF were “kainate selective glutamate receptor complex (GO:0032983)”, “lipid droplet (GO:0005811)”, and “dendrite (GO:0030425)”(Figure 6D,E).

### 3.5. Pathway Analysis

**Figure 7:**
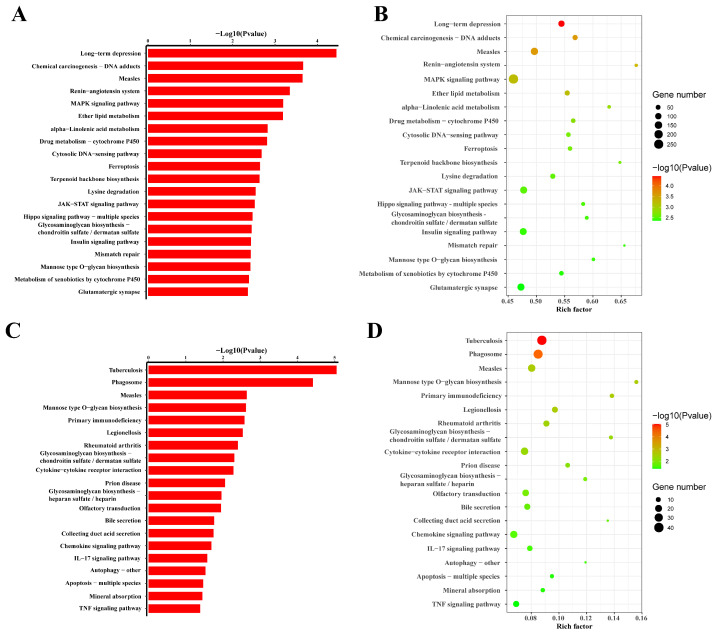
Pathway analysis of eccDNA-associated mRNAs: (**A**,**B**) refer to the down-regulated pathways of eccDNAs; (**C**,**D**) refer to the up-regulated pathways of eccDNAs.

By comparing eccDNAs of slimming grass carp with common grass carp, we identified the 378 upregulated pathways of eccDNA-related genes with different expressions, and 59 pathways were significant. These pathways included long-term depression, chemical carcinogenesis DNA adducts, measles, the renin–angiotensin system, MAPK signalling pathway, ether lipid metabolism, etc. There were 315 downregulated pathways found, of which 22 were significant, including tuberculosis, phagosome, measles, etc. (Figure 7).

### 3.6. Differential Expression Profile of eccDNAs in Slimming Grass Carp

When the differences in eccDNA expression in muscle between slimming grass carp and common grass carp were compared and examined, 16,022 up-regulated and 1216 down-regulated genes (|log2 FC| ≥ 1, *p* < 0.05) were found. Certain important genes—such as myosin heavy chain, myosin light chain, muscle segment homeobox C (*MsxC*), calsequestrin, calmodulin, voltage-sensitive calcium channels, and so on—that are involved in the functional regulation of muscle contraction and muscle fibre structure were found to be highly up-regulated within the differently expressed eccDNAs in slimming grass carp (Table 2). In the down-regulated genes, few genes were found to be correlated with muscle (with the exception of integrin and fibroblast growth factor), but they were mainly involved in physiological function, metabolism, and immune response, including Rap guanine nucleotide exchange factor, teneurin transmembrane protein-3, immunoglobulin C-2 Type, AIG1 family, and peptidase S1 gene.

## 4. Discussion

Since the first report in 1965 [20], eccDNA has been detected in a number of organisms, such as yeast, nematodes, ciliates, plants, and mammals. In yeast, about 23% of genetic information is from eccDNAs [10,23]. In humans, over 100,000 eccDNAs have been isolated from muscle and blood cells and found to contain a substantial amount of genetic information [30]. These findings imply that eccDNA might play an important role in the regulation of cellular physiological functions [15]. Currently, human tumours and diseases are the main focus of most eccDNA investigations [15,17,19,31], while some have started to concentrate on animal breeding as well [22,23]. There have been no reported eccDNA studies on aquatic animals, particularly fish. In this study, we first sequenced the eccDNAs from two kinds of grass carp with different muscle qualities: slimming grass carp and common grass carp. Their eccDNAs information and differences were generally compared and analysed, which was expected to provide a new eccDNAs research direction, as well as an analytical route by which to elucidate the mechanism of muscle character in fish.

In this study, a large number of eccDNAs—82,238—were found in slimming grass carp when compared to common grass carp. Møller et al. [30] detected more than 100,000 different eccDNAs from the muscle cells of men who undertook regular exercise and men who lived sedentary lifestyles. Møller et al. [23] characterised 300,000 eccDNA in the muscle tissue of three pigeons of different ages and with different flight behaviour, suggesting that eccDNA, as a DNA molecule independent of the chromosome, is also crucial for regulating cellular physiological functions. The cultivation process for slimming grass carp involves placing common commercial grass carp into clean flowing water without feeding them for about 2 months. This results in their weight loss and reduced fat, with no other treatment conducted during the process. Nonetheless, large amounts of eccDNAs were still detected, enriching in their muscle cells, revealing that the eccDNAs might be involved in the regulation linked to muscle character. Through GO analysis, we found that the genes were involved in the functions of cellular structure and response, cell immunology, enzyme activity, and so on. From the differential eccDNAs, we identified up-regulated genes mostly associated with muscle structure and contraction, including myosin heavy chain (*MHC*), myosin light chain (*MLC*), muscle segment homology box C (*MsxC*), calsequestrin, calmodulin, voltage-sensitive calcium channels, and so on. These findings suggest that in the muscle cells of slimming grass carp, the contents of eccDNAs correlated with muscle character regulation were mainly concentrated on the physiological function of muscle contraction, which, in turn, affected the quality of the meat. Different from our other eccDNA study on the crisp grass carp (unpublished), a grass carp which exhibited much higher muscle hardness and crispiness than the common grass carp and slimming grass carp, the increasing eccDNAs found in crisp grass carp were mainly associated with the functional regulation on muscle fibres, muscle development and differentiation, cytoskeleton, and extracellular matrix, including collagen, talin, catenin, troponin, calreticulinand cadherin, etc. The results, when compared, indicate that the two types of grass carp had distinct mechanisms for altering their muscle characteristics. Moreover, EccDNA may be crucial for functional regulation, and the change in meat quality in slimming grass carp may be impacted primarily by muscle contraction.

Myosin, the major protein of the contractile component of skeletal muscle cells, is a hexamer composed of two heavy chain subunits (MHC) and four light chain subunits (MLC) [32]. The C-terminal half of MHC is responsible for the thick filament formation of myosin. The N-terminal regions of MHC, together with MLC, form globular heads that contain ATPase and actin-binding sites and generate contractile force through hydrolysis of ATP and interaction with F-actin [33,34]. MHC engages in interactions with MLC and takes part in the regulation of myosin activity and muscle contraction. Previous studies revealed that the expression of *MHC* and *MLC* increased after certain movements or exercises. Wilborn et al. [35] reported that after a single bout of two resistance exercise intensities in untrained men, the mRNA expressions of all *MHC* isoforms increased. Willoughby and Nelson [36] also observed an evaluation of the *MHC* gene after a single session of heavy-resistance exercise in untrained individuals. Puhke et al. [37] concluded that during the following 54 weeks of strength and power training, the proportion of MLC1f and MLC2 in human vastus lateralis muscle increased. In this study, the eccDNA of *MHC* and *MLC* were up-regulated in the slimming grass carp, pointing out that during the slimming process, the stimulation of flowing water enhanced the exercise of grass carp; the eccDNA numbers of the two genes increased in the muscle cell, regulated and strengthened the muscle contraction, and thus altered the muscle character of the slimming grass carp and ultimately affected the meat quality. 

The *MsxC* (muscle segment homeobox C) gene belongs to the Hox gene family. Recent studies have demonstrated that the muscle segment homeobox C (*MsxC*) gene plays a crucial role in the regulation of muscle development. In Drosophila, the *MsxC* homologous gene muscle segment homeobox (*Msh*) is known to play an important role in myogenesis [38]. In both *Hemibarbus labeo* and zebrafish, *MsxC* is mainly expressed in the anterior part of the sarcomere; the development of the large lateral muscle can be directly impacted by disruption of the zebrafish *MsxC* gene [38,39]. Pang et al. [40] reveal that MsxC occurs in the differentiation of white and red muscle, and it has the ability to positively regulate white muscle and negatively regulate red muscle. In our study, eccDNAs of the *MsxC* gene in slimming grass carp were significantly increased, suggesting that the gene might have played a role in the regulation of muscle structure and development when the grass carp was under slimming cultivation treatment. 

Calcium is an element that is essential for numerous biological functions. The release of Ca^2+^ from the intracellular sarcoplasmic reticulum (SR) Ca^2+^ store contributes greatly to the contraction of skeletal muscle [41]. This study revealed a substantial upregulation in the eccDNA content of calsequestrin and calmodulin, which are related to calcium metabolism. The two genes both affect muscle contraction by regulating the amount of Ca2+. Calsequestrin is one of the most abundant proteins in the SR of skeletal muscle. It binds large amounts of Ca^2+^ within SR and concentrates it at the junctional face of the terminal cisternae near the sites of Ca^2+^ release [42,43,44]. This is critical for the ability of the SR to store Ca^2+^ and control the Ca^2+^ release. Calmodulin is a versatile Ca^2+^ sensor with high-affinity Ca^2+^ binding ability, mediating a variety of Ca^2+^-dependent signalling processes, including regulation of enzymatic or ion channel activities, synaptic transmission, and gene expression [45].

Additionally, several ion channel genes related to muscle contraction, including voltage-sensitive calcium channels (*VSCC*), dihydropyridine (DHP)-sensitive calcium channels, and voltage-dependent T-type calcium channel eccDNA, were also found to be up-regulated. These genes regulate the voltage sensitivity of Ca^2+^ channels, which enables cells to be excited in a short time and causes muscles to contract rapidly. Voltage-sensitive calcium channels can control the opening time of the Ca^2+^ channel in order to influence the membrane potential, thus regulating the contraction of skeletal muscle [46,47]. Dihydropyridine-sensitive calcium channels are abundant in skeletal muscle; they regulate the voltage sensitivity of Ca^2+^ channels on SR and cause muscle cells to excite over a relatively shorter time period to meet the demands of rapid contraction [48]. In muscle that is regenerating, voltage-dependent T-type calcium channels can induce myoblast fusion or myotube maturation [49]. The results of this study showed that the aforementioned genes were significantly upregulated in slimming grass carp, revealing that during the slimming cultivating process, Ca^2+^ channel and Ca^2+^ regulation genes related to muscle contraction were highly expressed, which affected the muscle contraction movement by alternating Ca^2+^ content and, in turn, impacted the muscle characteristic of the slimming grass carp.

Furthermore, certain eccDNAs were found to be down-regulated in slimming grass carp, such as Rap guanine nucleotide exchange factor, teneurin transmembrane protein-3, immunoglobulin C-2 Type, AIG1 family, and peptidase S1. These genes were significantly down-regulated and have little correlation with muscle structure but are mainly involved in the physiological function, metabolism, and immune response. In the cultivation process from common grass carp to slimming grass carp, common grass carp were starved for a period of time, with little food eating. As a result, a significant amount of fat was burned during the starvation treatment, which may have had an impact on the normal physiological metabolism and immune system of the fish but had no detrimental effects on their muscle structure. Thus, few genes related to muscle regulation were found to be down-regulated.

The above results show that compared with common grass carp, a large amount of muscle related to eccDNAs was enriched in the slimming grass carp muscle cells. The majority of genes are involved in the functional regulation of muscle contraction and structure, which indicates that during the slimming process, the influence was mainly concentrated on muscle contraction and movement, as well as structure. The basic method of slimming grass carp is placing common grass carp in clean water and starving them for approximately two months. The water’s flow also helps to strengthen the carps’ movements, which increases their consumption of fat and results in weight loss. During the process, no other stimulation and treatment is conducted. When they are hungry, common grass carp in the clean, flowing water just swim or move around freely. It is possible that such movement promotes the regulation of certain linked genes and causes the acceleration of muscle contraction. Despite transcriptome and proteomic research, no studies have been conducted recently that concentrate on the molecular mechanism of muscle character in slimming grass carp. In the present study, we analysed the genetic differences between common grass carp and slimming grass carp based on the eccDNA level, which was supposed to provide molecular evidence, as well as new research direction, to shed light on the mechanism underlying the muscle character of slimming grass carp.

## 5. Conclusions

In conclusion, we first reported the eccDNAs in fish and verified that eccDNAs participated in the regulation of the muscle firmness increase in grass carp. We found that massive eccDNA correlated with muscle character were enriched in the muscle cells during the muscle firmness increase from grass carp to slimming grass carp, including genes associated with muscle structure, contraction, muscle fiber, calcium metabolism, etc. All these characteristics indicate that the enriched eccDNAs might play a role in the activation of muscle firmness, increase regulation, and affect the meat character. Our preliminary data present a novel view for the study of the muscle-hardening mechanism at the eccDNA level and provide a new perspective and research direction for elucidating the regulation mechanism of muscle character in fish.

## Figures and Tables

**Table 1:** Raw reads, clean reads, and Q30 of samples.

Sample Name	Raw Reads	Clean Reads	Q30 (%)
Slimming grass carp 1	149,672,200	130,405,698	90.11
Slimming grass carp 2	185,402,510	164,979,168	91.25
Slimming grass carp 3	146,103,008	125,514,344	89.46
Common grass carp 1	157,244,712	139,871,412	91.78
Common grass carp 2	139,295,000	123,377,174	91.32
Common grass carp 3	153,561,782	130,942,792	91.82

**Table 2:** Differentially expressed eccDNA-associated genes involved in muscle contraction and muscle structure and development of slimming grass carp in comparison to common grass carp.

Gene Description	Annotation	Regulation	log2FC	*p*-Value
Muscle fiber structure and development				
Myosin heavy chain, fast skeletal muscle	GC01Gene03301	Up	21.2875216485768	0.000000051702
Myosin light chain, fast skeletal muscle	GC01Gene04413	Up	11.7632038868467	0.002605161394
Myosin binding protein C, fast type a	GC01Gene04318	Up	11.7632038868467	0.002605161394
Myosin, light chain kinase	GC01Gene04351	Up	11.7632038868467	0.002605161394
Myosin phosphatase	GC01Gene04201	Up	11.7632038868467	0.002605161394
Actin, alpha	GC01Gene16235	Up	21.2677854176002	0.00000005320
Capping protein	GC01Gene07223	Up	11.2325831506477	0.00404054634
Muscle segment homeobox C (MsxC)	GC01Gene17144	Up	21.0562052584647	0.000000072098
Integrin	GC01Gene09573	Down	−9.47427441877102	0.00925059453
Fibroblast growth factor	GC01Gene14267	Down	−9.47427441877102	0.00925059453
Calcium metabolism				
Calsequestrin	GC01Gene12461	Up	26.0132467521269	0.000000000027
Calmodulin	GC01Gene00579	Up	21.2677854176002	0.00000005320
Voltage-sensitive calcium channels	GC01Gene04287	Up	11.7632038868467	0.002605161394
Dihydropyridine (DHP) sensitive calcium channel	GC01Gene04863	Up	11.7632038868467	0.002605161394
Voltage-dependent T-type calcium channel subunit	GC01Gene00457	UP	8.55777414188946	0.017851975849

## Data Availability

The data generated and analyzed in this study are included within the article and are available from the corresponding author upon reasonable request.
